# Genomic prediction using a cost-effective mid-density SNP platform in spring wheat

**DOI:** 10.3389/fpls.2026.1892246

**Published:** 2026-07-20

**Authors:** Yaotian Gao, Guriqbal Singh Dhillon, Amandeep Kaur, Pabitra Joshi, Justin Wheeler, Jianli Chen

**Affiliations:** Department of Plant Sciences, University of Idaho Aberdeen Research and Extension Center, Aberdeen, ID, United States

**Keywords:** fixed effect, genomic selection, marker density, plant breeding, spring wheat, yield prediction

## Abstract

Genotyping cost is one of the major limiting factors for wide adoption of genomic selection in wheat. The present study compared two genotyping platforms, a low-density 4K single nucleotide polymorphism (SNP) chip and a Wheat iSelect 90K SNP, in genomic prediction of grain yield (YLD) and yield related traits in a training population composed of 224 spring wheat lines, and a new population composed of 141 spring wheat lines, with and without consideration of a fixed effect for major plant adaptation genes-derived competitive allele specific PCR (KASP) markers. Five models were evaluated and compared, which included 4K SNP-only, 4K SNP + KASP, 90K SNP-only, 90K SNP + KASP, and KASP-only. Within the training population, the 90K array showed slightly higher predictive ability for YLD, whereas the 4K panel performed better for thousand kernel weight (TKW). Incorporating KASP fixed effects, the 4K SNP + KASP and 90K SNP + KASP models significantly improved prediction for YLD, heading date (HD), and plant height (PHT) compared to the corresponding SNP-only models. KASP-only outperformed both SNP-only models for HD, but for PHT it only outperformed the 4K SNP-only model. In cross-population prediction, KASP-only models outperformed 4K SNP-only for HD and PHT. These results suggest that breeding programs can deploy genomic selection with a mid-density marker platform, especially, the 4K SNP + KASP model offers a cost-effective strategy for genomic prediction, while KASP-only genotyping provides a practical ultra-low-cost option for screening heading date and plant height.

## Introduction

Accelerating genetic gain in wheat is a practical priority for maintaining global food security, particularly under the budgetary and logistical constraints faced by modern breeding programs (Shiferaw et al., 2013; Erenstein et al., 2022; Food and Agriculture Organization (FAO) et al., 2026). Genomic selection (GS) provides an effective framework for this goal by enabling genome-wide marker data to reliably guide early selection decisions, thereby shortening breeding cycles and increasing selection efficiency (Meuwissen et al., 2001; Heffner et al., 2010; Crossa et al., 2017). Despite its methodological maturity, large-scale implementation of GS is constrained by the cost of genotyping thousands of breeding lines (Bassi et al., 2016; Rasheed et al., 2017). To address this limitation, two complementary strategies have been widely explored: 1) reducing per-line genotyping costs through lower-density marker panels (Norman et al., 2018; Kriaridou et al., 2023), and 2) increasing information efficiency by explicitly modeling major-effect loci as fixed effects (Sarinelli et al., 2019; Kim et al., 2022). Low-density SNP panels can achieve prediction accuracies comparable to high-density arrays once marker coverage exceeds the saturation threshold imposed by linkage disequilibrium (Meuwissen, 2009; Norman et al., 2018). In parallel, modeling known adaptation genes as fixed effects substantially improves prediction accuracy for traits influenced by large-effect loci, particularly in wheat (Sarinelli et al., 2019; Gao et al., 2025).

Recent studies have established the methodological validity of this fixed-effect approach by showing consistent gains for key adaptive traits when major genes are explicitly incorporated (Combs and Bernardo, 2013; Michel et al., 2016; Sehgal et al., 2020; Gao et al., 2025). However, this strategy has yet to be widely deployed in breeding pipelines (Robertsen et al., 2019), since it is unclear how prediction accuracy depends on background marker density, under what conditions low-density panels can approximate high-density performance, and how robust fixed-effect gains are across populations (Hickey et al., 2014; Norman et al., 2018; Sarinelli et al., 2019). These questions are especially important for breeding programs that must balance prediction accuracy against genotyping cost across multiple breeding cycles.

To address these gaps, we evaluated two spring wheat panels, GP502 as a training population and GP503 as an independent validation population, to systematically examine interactions among marker density, fixed-effect integration, and prediction context. We benchmarked a cost-effective 4K SNP panel against a 90K SNP array, assessing performance both within and across populations. For traits with known adaptive genetic architectures, this design allowed us to assess the optimum stage in the breeding cycle to integrate a limited number of KASP markers as fixed effects. Heading date (HD) and plant height (PHT) were targeted due to their well-characterized control by major developmental genes.

The study was structured around three objectives: 1) define scenarios in which a low-density 4K panel can effectively substitute for a high-density 90K array, 2) identify conditions under which KASP fixed effects deliver maximal gains, and 3) assess the transferability of these effects across populations. To isolate the marginal contribution of each component, we compared three model classes: SNP-only, KASP-only, and combined SNP+KASP incorporating fixed effects. This comprehensive design evaluates trait-specific trade-offs between marker quantity (SNP arrays) and biologically informative marker structure (KASP markers). We assess these trade-offs both within a training population and in the more challenging cross-population prediction context. To our knowledge, this study provides the first direct comparison in spring wheat between the predictive performance of KASP-only models and high-density SNP arrays, offering practical guidance for strategic genotyping decisions in breeding programs.

## Materials and methods

### Plant materials

Plant materials consisted of two spring wheat panels, GP502 and GP503, representing distinct but related breeding populations. GP502 was used as the training population for model development, while GP503 served as an independent validation population. The GP502 panel was composed of 224 spring wheat varieties and elite lines (**Supplementary Table 1**). The collection was developed by breeding programs in the Pacific Northwest region of the United States and the International Maize and Wheat Improvement Center (CIMMYT, Mexico City, Mexico). It encompasses soft white, hard white, and hard red market categories of spring wheat cultivated in the Americas. Most of the lines have been used in regional variety development programs. This population has been described in a previous study (Gao et al., 2025). The GP503 panel consisted of 141 elite lines selected from the University of Idaho wheat breeding program (**Supplementary Table 2**).

### Phenotypic evaluation

Field trials were conducted across multiple environments to capture environmental variability relevant to breeding conditions. The trials were conducted at Aberdeen, Idaho (42°56’36” N, 112°50’22” W) under irrigated conditions from 2022 to 2024. Trials were arranged in a randomized complete block design with two replications per environment. Each genotype was planted in 3.0 m plots composed of seven rows with 21 cm spacing. Individual plots were treated as experimental units. Standard agronomic management practices were applied across all plots to minimize non-genetic variation (Marshall et al., 2019). Management practices, soil fertility, and environmental conditions varied across the years (**Supplementary Table 3**).

HD was recorded as the Julian date (days from January 1st) when 50% of the plants had spikes emerging from the flag leaf sheath. PHT was measured in centimeters from the soil surface to the tip of the spike, excluding awns. Thousand-kernel weight (TKW) was estimated by weighing 100 randomly selected seeds per plot. Grain yield (YLD) was obtained by mechanically harvesting plots and adjusting grain weight to a standardized moisture content.

### Phenotypic data analysis

The analysis integrated replications through random effects linear models across different environments, employing META-R version 6.4 (Alvarado et al., 2020). Phenotypic data were analyzed using linear mixed models to estimate best linear unbiased predictors (BLUPs) for each trait. Genotype was treated as a random effect, while environment was modeled as a fixed effect. Broad-sense heritability was estimated on an entry-mean basis using variance components derived from the mixed model.

### Genotyping and quality control

The populations were genotyped using two distinct platforms. For the GP502 population, genotyping was performed with Illumina’s 90K iSelect SNP chip. The USDA/ARS Cereal Crops Research Unit in Madison, WI, supplied the raw data, which was then analyzed in Genome Studio v2.0.5 (Illumina, 2010). In this initial step, polymorphic markers were identified by distinct clustering and a minimum distance of 0.20 between the polar coordinates of normalized theta intensities. Concurrently, both GP502 and GP503 populations underwent a 4K Genotyping-by-Sequencing (GBS) analysis, conducted by the Deven See Lab at Washington State University (**Supplementary Tables 5**, **6**).

Following the initial genotyping, markers from both datasets were subjected to a quality control process using TASSEL v5.2.89 (Bradbury et al., 2007). Markers with either more than 10% missing data, or a minor allele frequency (MAF) below 5%, were excluded. This filtering resulted in 5,194 high-quality SNP markers (**Supplementary Table 4**) and 2,600 GBS markers (**Supplementary Tables 5**, **6**), which were used for all further analyses. A chromosome density plot was generated with SRplot to visualize the marker distribution (Tang et al., 2023).

### Genomic relationship matrix computation and comparison

All analyses were based on a common set of 224 individuals from the GP502 training population, genotyped with both the 4K GBS method and the 90K SNP array. For each set of genotype data, marker-to-chromosome assignment files were used to partition markers by sub-genome (A, B, and D). Markers with unknown (“Un”) chromosome assignments were excluded from sub-genome-specific analyses. GRMs were computed with A.mat from the rrBLUP package, which implements the VanRaden (2008) Method I formulation (centered by allele frequency and scaled by (2*p*(1 − *p*)) (VanRaden, 2008; Endelman, 2011). This calculation was performed separately for the entire genome and for the A, B, and D sub-genomes individually. To compare *K*_4_*_K_* and *K*_90_*_K_* for the whole-genome and each sub-genome, the correlation between the GRMs from the two arrays was then quantified using a Mantel test with 9,999 permutations via the mantel.rtest function from the ade4 package (Mantel, 1967; Dray and Dufour, 2007).

To illustrate the Mantel test findings, scatter plots of the pairwise GRMs (*K*_4_*_K_* and *K*_90_*_K_*) were generated, with a linear regression line and the Mantel *r* correlation coefficient overlaid. Additionally, to visualize the magnitude of differences between the GRMs, a heatmap of the whole-genome difference matrix (*K*_90_*_K_* - *K*_4_*_K_*) was generated using the pheatmap package with a divergent red-white-blue palette (Kolde, 2015).

### Principal component subspace comparison

The geometric congruence of the principal component (PC) spaces derived from each array was assessed using a second analysis pipeline. First, genotype matrices were standardized for PCA using VanRaden’s Method II (VanRaden, 2008). This method, which standardizes each marker by its own variance (2*p_j_*(1 – *p_j_*)), was chosen because it makes the contribution of each marker more even. This approach allows for a direct comparison of the PC subspaces defined by rarer variants, which are hypothesized to differ between the low-density (4K) and high-density (90K) arrays. This analysis complements the GRM comparison, which relies on VanRaden’s Method I and is more heavily influenced by common alleles. For this standardization, markers were filtered to remove monomorphic sites (*p* = 0 or *p* = 1), and genotypes (coded as 0, 1, 2) were standardized using the formula $Z=\left(X_{j}-2p_{j}\right)/\sqrt{2p_{j}\left(1-p_{j}\right)}$, where *p_j_* is the allele frequency of the *j*-th marker. Missing genotypes were imputed with 2*p_j_* before standardization. PCA was then performed on the standardized *K*_4_*_K_* and *K*_90_*_K_* matrices using the “prcomp” function from stats R package to extract PC scores (R Core Team, 2025). The similarity between PC score configurations was evaluated using Procrustes analysis via the “procrustes” function from vegan R package, where the *S*_4_*_K_* space was rotated and scaled to fit the *S*_90_*_K_* reference space (Dixon, 2003). Significance was determined with a permutation test using the protest function from vegan R package (9,999 permutations) yielding a *t*_0_ statistic. To formally quantify subspace similarity, principal angles (*θ*) were calculated by obtaining orthonormal bases (*Q*_4_*_K_*, *Q*_90_*_K_*) via the qr function from base package, followed by a Singular Value Decomposition using the svd function from base package on the cross-product matrix $\left(Q_{4K}^{T}Q_{90K}\right)$. The resulting singular values (*d_i_*) were used to calculate angles as $\theta_{i}=\arccos\left(d_{i}\right)\;\times 180/\pi$. The overall subspace distance was summarized using the Grassmannian chordal distance $d_{c}=\sqrt{\sum_{i=1}^{k}\sin^{2}\left(\theta_{i}\right)}$ (Björck and Golub, 1973; Edelman et al., 1998). These subspace comparisons were performed iteratively for the top 10 principal components.

### Sub-genome variance component analysis

We partitioned the total genetic variance among the A, B, and D sub-genomes using the same 224 individuals and the previously defined sub-genome marker sets. Instead of the additive GRMs used for the Mantel test, we constructed sub-genome-specific relationship kernels under a Reproducing Kernel Hilbert Space (RKHS) framework (Gianola and Van Kaam, 2008).

For each platform (4K and 90K) and each sub-genome (A, B, D), the genotype matrix *X* was mean imputed at the marker level (2*p_j_*) and then standardized (centered and scaled). Pairwise squared Euclidean distances ( $d_{ij}^{2}$) were computed and converted to a Gaussian (RBF) kernel *K_raw_* with elements $K_{raw,\;\;ij}=exp\left(-hd_{ij}^{2}\right)$, where the bandwidth used the median heuristic $h=1/\text{median}\left(d_{ij}^{2}\right)$ (Garreau et al., 2017). This initial kernel was then double-centered and scaled to create the final kernel *K_k_* used for analysis: $K_{k}=HK_{raw}H,\;\;H=I-11^{⊤}/\text{n}$. The final kernel *K_k_* was scaled such that $tr\left(K_{k}\right)/n=1$. This process resulted in six distinct kernels $\left(K_{4K-A},\;K_{4K-B},K_{4K-D},K_{90K-A},K_{90K-B},K_{90K-D}\right)$.

For each trait (HD, PHT, TKW, YLD) and platform, we fitted an independent multi-kernel Bayesian mixed model. The model was defined as:


$$y=g_{A}+g_{B}+g_{D}+e$$

Where *y* is the vector of phenotype BLUPs, *g_A_*, *g_B_*, and *g_D_* are the random genomic effects for the A, B, and D sub-genomes and assumed to follow $g_{k}\sim N\left(0,\;K_{k}\sigma_{k}^{2}\right)$, and *e* is the residual error vector assuming $e\sim N\left(0,\;I\sigma_{E}^{2}\right)$. *K_k_* represents the corresponding RKHS kernel for sub-genome *k*. The model was implemented using the BGLR R package, running for 10,000 iterations with a 2,000-cycle burn-in to estimate the variance components for each sub-genome (Pérez and de Los Campos, 2014). The use of multiple kernels to partition genomic signal by sub-genome follows established kernel-based genomic prediction practice (Morota and Gianola, 2014).

For the assessment of the relative contribution of each sub-genome to the total genetic variance, the genetic variance (*Var_Genetic_*) was defined as the sum of the three sub-genome variance components:


$$Var_{Genetic}=\sigma_{A}^{2}+\sigma_{B}^{2}+\sigma_{D}^{2}$$

The proportional contribution, or “Share,” of each sub-genome was then calculated as its variance divided by this total genetic variance (e.g., $Share_{A}=\sigma_{A}^{2}/Var_{Genetic}$).

### Major genes KASP markers

KASP markers targeting major genes controlling flowering time and PHT were genotyped. These markers included loci previously shown to have large and stable effects across spring wheat backgrounds. KASP genotypes were coded additively according to allele dosage for use in genomic prediction models. Detailed information on each marker, including the specific alleles assayed and their known functional effects, is provided in **Supplementary Table 7**.

### Statistical models for genomic prediction

Genomic prediction was performed using genomic RKHS, which employed a Gaussian (RBF) kernel as in the variance-component analysis (Gianola et al., 2006). SNP-only models (RKHS) used genome-wide marker information without explicit modeling of major loci. SNP + KASP Models (RKHS + Fixed Effects) incorporated KASP markers as covariates to account for major gene effects explicitly. KASP-only models were constructed using KASP markers as the sole predictors.

### Prediction accuracy evaluation

Prediction accuracy was calculated as the Pearson correlation between observed phenotypic BLUPs and genomic estimated breeding values. Within-population prediction accuracy was assessed using repeated 5-fold cross-validation within GP502. Cross-population prediction accuracy was evaluated by training models in GP502 and validating predictions in GP503. All analyses were conducted using standardized statistical pipelines to ensure comparability among model types.

### Learning curve analysis

Learning curves were generated using a stratified, nested sampling strategy, which was performed independently for the 4K and 90K platforms to evaluate prediction accuracy as a function of marker density.

First, to reduce marker redundancy, the full SNP datasets were pruned based on linkage disequilibrium (LD) using the “snpgdsLDpruning” function from the “SNPRelate” R package (Zheng et al., 2012). This procedure utilized a 500 kb sliding window and an *r*^2^ threshold of 0.80 to create a representative pool of markers.

From this LD-pruned pool, a series of nested marker subsets (where $N_{1}\subset N_{2}\subset \ldots \subset N_{k}$) was selected to ensure monotonic learning curves and reduce Monte-Carlo variability across marker-set sizes. This sampling was stratified by the A, B, and D wheat genomes to maintain proportional coverage. For the 4K platform, nested subsets of *N* = {100, 500, 1000, 1500, 2000} markers were created. For the 90K platform, nested subsets of *N* = {100, 500, 1000, 1500, 2000, 2500, 3500} were generated. This entire stratified sampling process was repeated 10 times to create 10 independent sampling replicates. Model performance was evaluated using a 5-fold cross-validation (CV) scheme. This 5-fold CV was repeated 50 times. For a given trait, the set of 50 CV-fold assignments was pre-computed and identically applied across all analyses. This pre-computation ensured that both models, at every marker density *N* on both platforms, were compared using the same training and testing sets.

For each marker subset *N*, the previously described “SNP-only” (RKHS) and “SNP+KASP” (RKHS + Fixed KASPs) models were fitted. This analysis was run using the BGLR package with 10,000 iterations and a 2,000-iteration burn-in.

For the final statistical summary, accuracies were first averaged across the 5 folds, then averaged across the 50 CV replicates. This process resulted in one robust accuracy estimate for each of the 10 sampling replicates. The final learning and gain curves report the mean and standard error (SE) calculated across these 10 sampling replicates, with the sampling replicate serving as the unit for statistical inference.

Prediction accuracy was defined as the Pearson correlation coefficient (*r*) between the predicted genetic values and the observed phenotypic BLUPs in the test set. The “Gain” from KASP markers was calculated as the simple difference in accuracy


$$Gain=r_{SNP+KASP}-r_{SNP-only}$$

### Assessing the predictive contribution of sub-genomes

A comprehensive CV scheme was implemented to assess the genomic prediction performance for TKW, focusing specifically on quantifying the distinct predictive contributions of the A, B, and D sub-genomes. This analysis used the Gaussian kernels previously described, which were derived from both the 4K and 90K marker platforms. We compared the predictive ability of four distinct model configurations for each platform. These included a model using the complete kernel derived from all genomes, and three separate models using kernels derived exclusively from the A, B, or D genomes.

A 5-fold cross-validation design was established and repeated 50 times for statistical robustness. To ensure a direct and fair comparison, a paired cross-validation design was used. For each of the 50 repetitions, a single, unified set of 5-fold indices was randomly generated. This partitioning of training and testing sets was then used to evaluate the four model configurations across both marker platforms. This paired approach ensured that any observed differences in accuracy are attributable to the kernel/model configuration rather than random variation in data partitioning.

All genomic prediction models were implemented using the RKHS model setting within the R package BGLR. For each CV fold, the model was trained on the training set data to predict the genomic-estimated breeding values (GEBVs) of the individuals in the test set. The Gibbs sampler was run for 10,000 iterations, with the first 2,000 iterations discarded as burn-in. Prediction accuracy for each repetition was then calculated as the Pearson’s correlation coefficient between the observed phenotypic values and the predicted GEBVs for the held-out test sets.

### Statistical analysis of variance components and residual genomic selection

A three-stage analysis was performed using R to quantify the impact of major genes (KASP markers) on genomic variance. First, we quantified the reallocation of genomic variance by comparing a SNP-only model (M0) with a SNP+KASP model (M1). The M0 model, representing RKHS-only effects, was fitted using the BGLR package. This model used a Radial Basis Function (RBF) kernel, *K*, to estimate the genomic variance component ( $Var\left(U_{M0}\right)$). We then calculated the relative “shrinkage” or drop in genomic variance explained by the RKHS kernel after accounting for the KASP markers fitted as fixed effects in M1. This variance reallocation was quantified as the percentage drop ( $\frac{Var\left(U_{M0}\right)-Var\left(U_{M1}\right)}{Var\left(U_{M0}\right)}$), where $Var\left(U_{M1}\right)$ represents the RKHS variance component from the M1 model.

Second, we evaluated the accuracy of “Residual Genomic Selection (GS)” to quantify the predictability of the polygenic background after accounting for the effects of major KASP markers. To prevent data leakage, the analysis was rigorously nested within the CV scheme previously described. For each CV replicate and fold, we first fitted a fixed-effect linear model using only the training set phenotypes as the response and the corresponding KASP markers as predictors. The residuals from this training model were then immediately extracted and used as the adjusted response variable to train the RKHS-GS model. Concurrently, the linear model, which was fitted only on the training data, was applied to the test-set KASP markers to predict their phenotypic contribution. These predictions were subtracted from the observed test set phenotypes to calculate the “true” test set residuals. The final accuracy for that fold was computed as the Pearson correlation between the GS model’s predictions and these “true” test set residuals.

Third, we investigated the “conditional relevance” of population structure by quantifying the proportion of PC-explained variance that was redundant with the KASP markers. For each trait, we first computed the top ten principal components (PCs) from the genomic marker data (4K or 90K). We then calculated two coefficients of determination (*R*^2^), the $R_{y}^{2}$, derived from regressing the original phenotype onto these PCs ( y ~ PCs), the $R_{resid}^{2}$, derived from regressing the phenotypic residuals onto the same PCs ( residuals ∼ PCs). These residuals were obtained by fitting a linear model of the KASP markers on the original phenotype ( $residuals=y-\;{\hat{y}}_{KASP}$). Finally, the “PC-explained *R*^2^ drop” was calculated as $\frac{\left(R_{y}^{2}-R_{resid}^{2}\right)}{R_{y}^{2}}$, representing the proportion of the population structure’s explanatory power that was already captured by the fixed-effect KASP markers.

### Assessment of KASP marker effect stability

The stability and consistency of KASP marker effects were evaluated using three analyses. First, we assessed within-population model stability using the GP502 training set within a 5-fold CV framework, which was repeated 50 times. For each of the 50 replicates, the data was partitioned into 5 folds. A KASP-only linear model was trained five separate times, each time using four folds as the training partition and standardizing the genotype data based on the mean and standard deviation of that specific partition. This process yielded five distinct marker effect *β* vectors for each replicate. To quantify stability, we calculated the mean pairwise cosine similarity. This function operates by taking the resulting *p*×5 matrix of *β* vectors and iterating through all 10 unique pairs of columns (e.g., Fold 1 vs. Fold 2, Fold 1 vs. Fold 3,…, Fold 4 vs. Fold 5). For each pair, it computes the cosine similarity, defined as the dot product of the two vectors divided by the product of their L2-norms after removing non-finite values. The final stability metric for the replicate was the arithmetic mean of these 10 pairwise similarity scores.

Second, we evaluated the stability of the training model used for cross-population prediction by applying a bootstrap procedure to the GP502 training data. This analysis involved 1,000 iterations, where each iteration consisted of sampling the GP502 population with replacement to create a new training set. A KASP-only linear model was fit to this bootstrapped sample using only markers common to both populations, with genotypes standardized based on the mean and standard deviation of the bootstrapped sample itself. This process generated 1,000 *β* vectors, which were compiled into a *p*×1000 matrix. The same function was then applied to this matrix to calculate the average cosine similarity among all 1,000 *β* vectors, quantifying the stability of effect estimation from the training data.

Third, to measure the conservation of marker effect *ranks* between the training (GP502) and testing (GP503) populations, we computed the Spearman’s rank correlation (ρ) of their independently estimated *β* vectors. For this analysis, a linear model was first trained on the entire GP502 training set (TR), using GP502-specific standardization, to estimate a single vector *β_TR_*.* A* separate linear model was then trained on the entire GP503 test set (TE), using GP503-specific standardization, to estimate *β_TE_*. Finally, the Spearman’s rank correlation was computed between the *β_TR_* and *β_TE_* vectors to assess the consistency of marker effect ranking across the two distinct populations. This analysis is descriptive and not used for model selection or prediction tuning.

### Estimation of effective independent markers

The effective number of independent markers (*M_e_*) was estimated for the 4K and 90K genotyping platforms using a custom R script. The analysis began by loading the genotype matrices and their corresponding chromosome annotation files for each platform. Markers assigned to unanchored scaffolds were excluded from the analysis. We defined the effective number of independent markers (*M_e_*) as the participation ratio of the LD correlation matrix. For each chromosome, missing genotypes were imputed with the mean genotype (2*p_j_*), then markers were mean-centered and scaled by the sample standard deviation (variance = 1) to construct a marker-by-marker correlation matrix (*R_chr_*) (unit diagonal). We computed the squared Frobenius norm ( ${∥R_{chr}∥}_{F}^{2}=tr(R_{chr}^{2}$) and summed across chromosomes, while recording the total number (M) of non-monomorphic markers. Finally,


$$M_{e}=\;\frac{M^{2}}{\sum_{chr}{∥R_{chr}∥}_{F}^{2}}={\frac{\left(\sum_{i}\lambda_{i}\right)}{\sum_{i}\lambda_{i}^{2}}}^{2}$$

Where λ*_i_* are the eigenvalues of (*R*). This measure is equivalent to eigenvalue-based effective test number approaches (Nyholt, 2004; Li and Ji, 2005; Gao et al., 2008; Galwey, 2009) and provides a robust approximation to the independent information content of the panel.

### Fisher’s exact test for KASP genotype-distribution differences

We applied the Fisher–Freeman–Halton exact test for 2×3 contingency tables to assess whether genotype distributions differed between the two populations (GP503 vs. GP502) at each bi-allelic marker. For each marker, we formed a table with rows for population (GP503, GP502) and columns for the three genotype categories (Allele 1, Allele 2, and heterozygotes). The null hypothesis was equality of genotype distributions across populations and the alternative was that they differ. Two-sided p-values were computed under the multivariate hypergeometric model, conditional on the observed row and column margins. We used Monte Carlo sampling to approximate the exact p-value (two-sided), ensuring a sufficiently large number of replicates and a fixed random seed for reproducibility. Missing genotype calls were excluded. Analyses were conducted in R.

## Results

### Phenotypic variation

**Figure 1:**
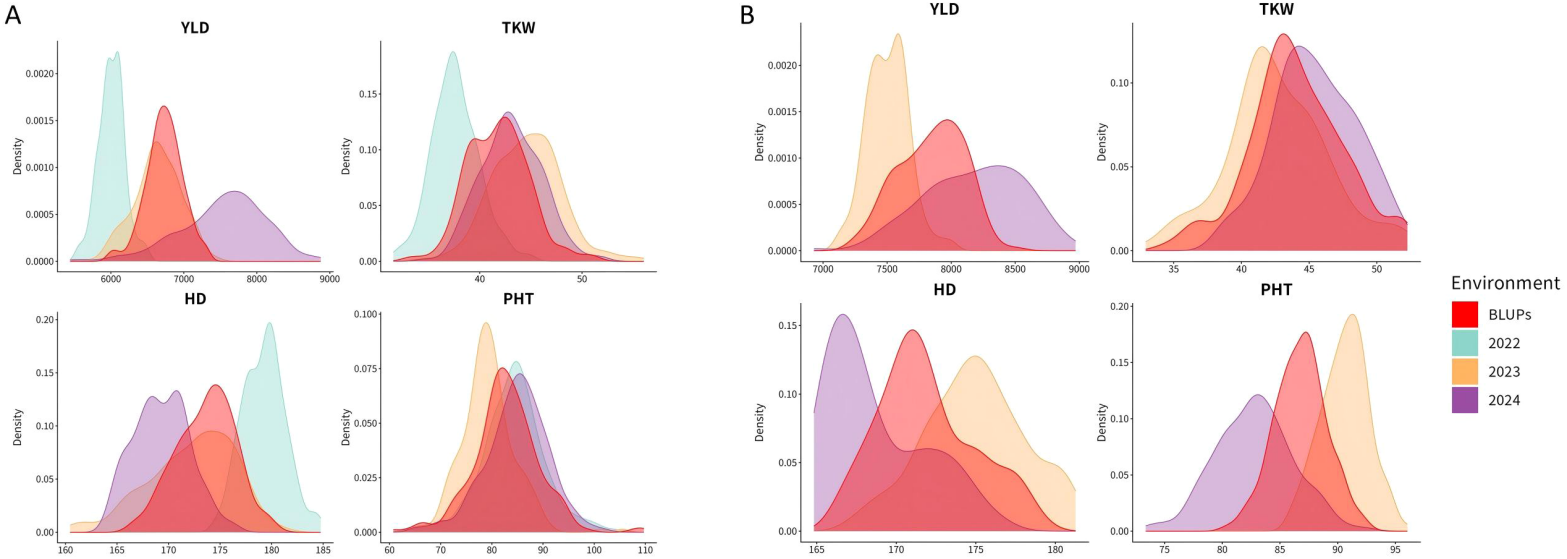
Kernel density curves for four traits in two spring wheat populations across years. **(A)** GP502 (training; 2022–2024) and **(B)** GP503 (testing; 2023–2024). Overlays show per-year distributions and the across-environment BLUPs for each trait: yield (YLD), thousand-kernel weight (TKW), heading date (HD), and plant height (PHT). Curves highlight inter-annual shifts (e.g., higher YLD in 2024 for both populations) and relative dispersion by trait (HD most stable; TKW/PHT more variable). Exact summary statistics (means, ranges, coefficients of variation) are provided in **Supplementary Table 8**.

Phenotypic evaluation revealed inter-annual fluctuation of agronomic traits in both the training (GP502) and testing (GP503) panels. In the training population (GP502), while heading date remained the most stable trait across environments, other agronomic traits, including PHT, TKW, and YLD, exhibited wider dispersion and significant inter-annual fluctuations (**Figure 1A**, **Supplementary Table 8**). For instance, grain yield showed pronounced environmental sensitivity, with mean values differing by over 1,500 kg/ha between extreme years. Similar phenotypic trends were observed in the testing population (GP503) (**Figure 1B**, **Supplementary Table 8**). TKW exhibited the highest relative dispersion, followed by YLD and PHT, while HD again proved to be the most stable trait with the lowest coefficient of variation.

Comparing the two populations, the validation set (GP503) demonstrated a distinct phenotypic profile characteristic of intensive breeding selection. Relative to the diverse training panel, the elite GP503 lines were characterized by higher YLD and TKW based on BLUPs. These phenotypic shifts between the diverse training set and the elite target population provide a rigorous testing ground for assessing the transferability of genomic prediction models.

### SNP marker distribution and genome coverage

The 4K GBS panel provided broad coverage of all 21 chromosomes with 2,600 high-quality SNPs (**Supplementary Table 9**, **Supplementary Figure 1**). Marker density followed the characteristic hierarchy of hexaploid wheat diversity, being highest in the B sub-genome (1,129 SNPs) and lowest in the D sub-genome (425 SNPs). These patterns were concordant with the 90K array, indicating that the low-density panel successfully captures the representative genomic landscape despite its reduced marker count (**Supplementary Table 10**).

### Concordance of population structure between 4K and 90K marker panels

The consistency between the low-density 4K and high-density 90K marker panels was evaluated through a multi-faceted comparison of the genetic relationship matrices and the resulting population structures they described.

**Figure 2:**
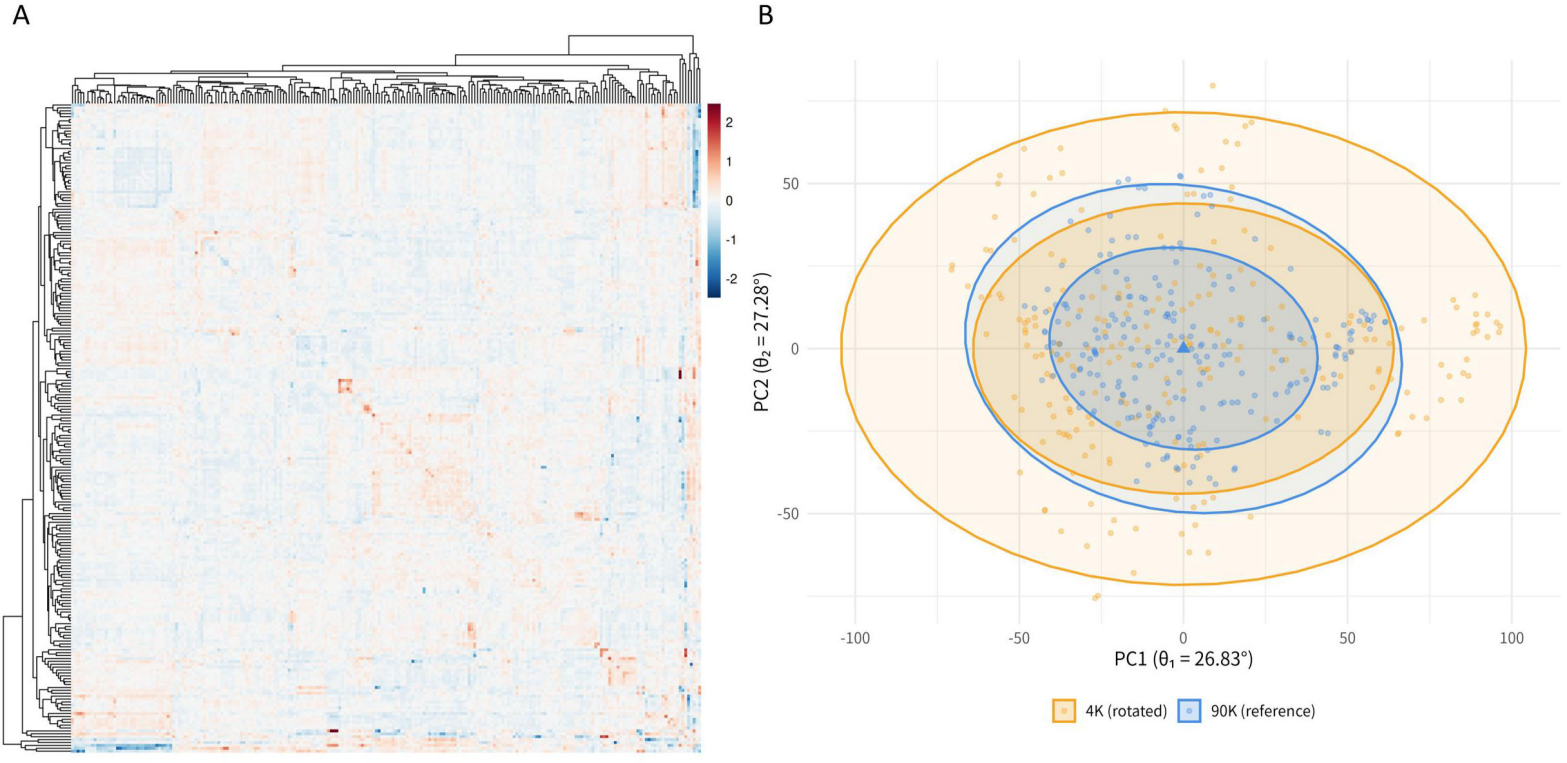
Comparison of population structure between 4K and 90K marker panels. **(A)** Differential heatmap of kinship matrices between the 4K and 90K panels. Red indicates higher kinship in 4K, blue indicates higher kinship in 90K, and light shades near zero indicate high similarity. **(B)** Overlay of the first two principal components (PC1 and PC2) from Procrustes analysis, showing the 68% and 95% confidence ellipses for 4K (rotated) and 90K (reference) panels. The rotation angles for PC1 (θ_1_) and PC2 (θ_2_) are indicated.

First, we generated a differential heatmap to visually inspect the element-wise differences between the two kinship matrices (**Figure 2A**). The heatmap was predominantly characterized by colors representing near-zero values, indicating a high degree of overall similarity. Minor to moderate, block-like deviations were observed, primarily concentrated along the diagonal within specific pedigree clusters. This pattern indicates high consistency between the two platforms, with minor deviations observed only among closely related individuals.

Next, we performed a Procrustes analysis to quantitatively compare the geometric configuration of the population as defined by the first 10 principal components (PCs) from each dataset. The Procrustes analysis yielded a correlation of r = 0.671 (*p<* 0.001), confirming that the population structures defined by the two panels are significantly correlated. Although the correlation magnitude reflects the expected resolution gap between 4K and 90K densities, the geometric alignment remains robust: the confidence ellipses are congruent, and centroids are coincident (**Figure 2B**). The observed rotation (*θ* ≈ 27°for PC1 and PC2, **Supplementary Table 11**) and the moderate correlation suggests that the 4K panel captures the same fundamental population stratification as the high-density array, albeit with higher residual variance (visualized as the larger ellipse).

Finally, the consistency between the kinship matrices was statistically validated using the Mantel test. A statistically significant correlation (*r* = 0.712, *p* < 0.001) was found between the whole-genome (ABD) kinship matrices derived from the 4K and 90K marker sets (**Supplementary Figure 2**). This correlation persisted across sub-genomes with correlations ranging from *r* = 0.678 in the A sub-genome to *r* = 0.571 in the sparser D sub-genome. Despite these variations, all correlations remained highly significant (*p* < 0.001), confirming that the 4K panel captures the fundamental relatedness structure.

Taken together, these analyses demonstrate that, given the linkage disequilibrium structure of the current population, the 4K panel is sufficient to reconstruct a kinship structure that is statistically comparable to that derived from the high-density 90K panel at both the whole-genome and sub-genome levels.

### Effect of marker density on sub-genome variance partitioning

**Figure 3:**
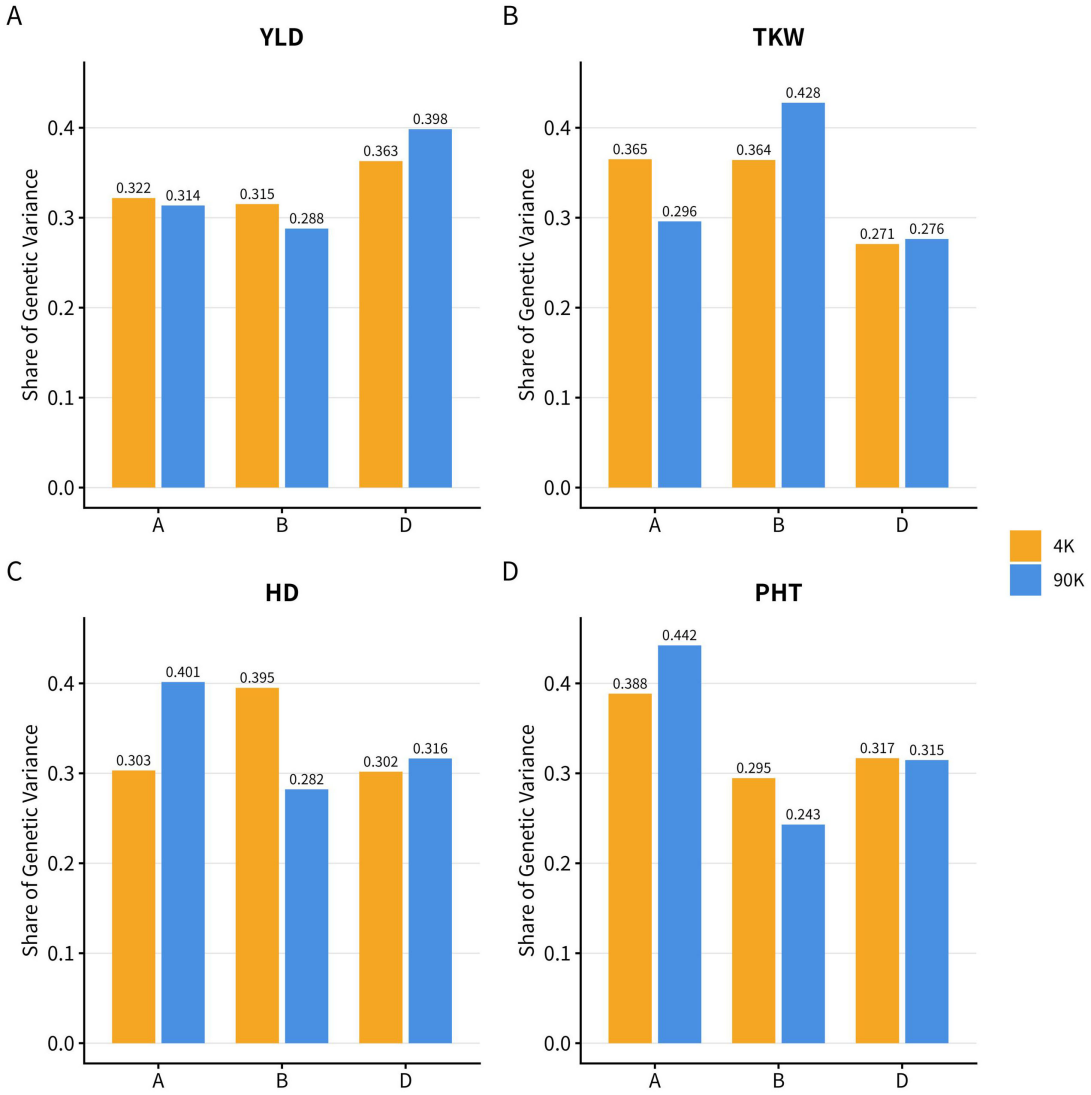
Effect of marker density on sub-genome variance partitioning for different agronomic traits. The figure illustrates the estimated proportion of genetic variance attributed to the A, B, and D sub-genomes using the 4K (orange) and 90K (blue) marker panels. Each panel represents a different agronomic trait: **(A)** YLD (Yield), **(B)** TKW (Thousand-kernel weight), **(C)** HD (Heading date), and **(D)** PHT (Plant height). Variance components were estimated using a multi-kernel RKHS model.

Variance partitioning analysis revealed generally consistent sub-genome contributions across marker densities, with notable exceptions for specific traits (**Figure 3**). For YLD and PHT, both panels assigned the largest proportion of genetic variance to the same sub-genomes (D and A, respectively), indicating stable signal capture. However, for TKW and HD, the 90K panel resulted in a distinct reallocation of genetic variance signals among sub-genomes compared to the 4K baseline. This discrepancy suggests that the increased marker density in the 90K array alters the weighting of sub-genome specific signals, potentially explaining the divergence in predictive ability observed for these traits.

### Genomic prediction accuracy within and across populations

**Figure 4:**
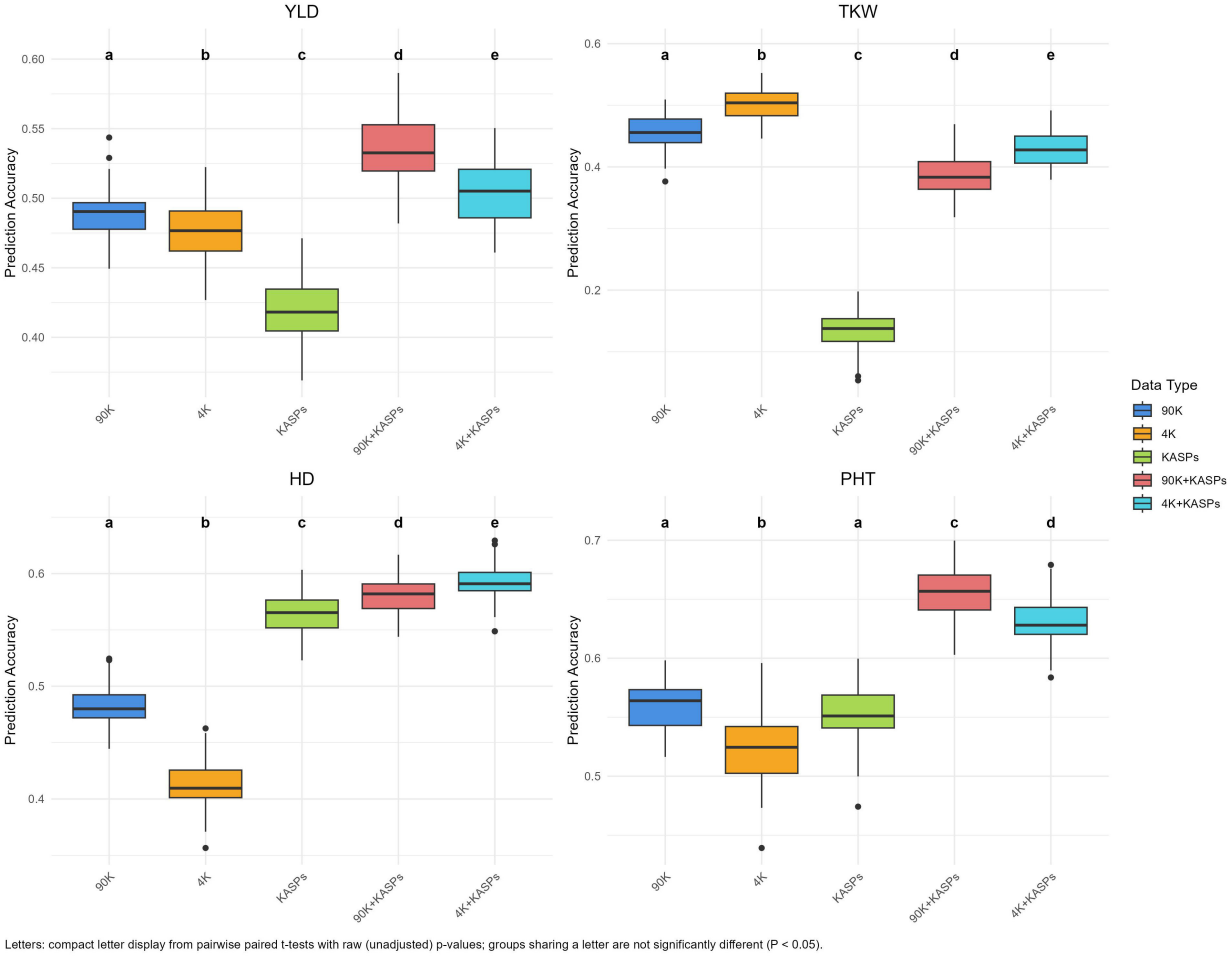
Genomic prediction accuracy of five marker models for four agronomic traits. Distribution of genomic prediction accuracy (Pearson’s correlation coefficient, *r*) from 50 replications of a 5-fold cross-validation for Grain Yield (YLD), Thousand-Kernel Weight (TKW), Heading Date (HD), and Plant Height (PHT). The five models compared are: 4K (baseline 4K SNP panel), 90K (baseline 90K SNP panel), KASPs (KASP markers only), 4K+KASPs (4K panel with all KASP markers as fixed effects), and 90K+KASPs (90K panel with all KASP markers as fixed effects). Boxes represent the interquartile range (IQR), the central line indicates the median, and whiskers extend to 1.5 times the IQR. Different uppercase letters indicate significant differences (P< 0.05) based on paired t-tests; models sharing a common letter are not significantly different.

Within-population prediction showed trait-dependent differences between the 4K and 90K SNP panels (**Figure 4**; **Supplementary Tables 12**, **13**). While trait-specific fluctuations were observed, such as a statistical advantage for the 90K panel in HD and PHT versus a performance advantage for the 4K panel in TKW, the absolute differences in predictive ability were moderate (**Supplementary Table 13**). These results demonstrate that the low-density 4K panel captures most of the relevant genetic variation, indicating diminishing returns from increasing marker density beyond the 4K baseline.

Incorporation of KASP markers as fixed effects resulted in a statistically significant increase in prediction accuracy for HD under both SNP densities (**Figure 4**, **Supplementary Table 12**). PHT prediction accuracy also increased significantly following the inclusion of KASP fixed effects. The relative improvement in prediction accuracy from KASP fixed effects was greater for the 4K panel than for the 90K array. In contrast, the inclusion of KASP fixed effects resulted in only modest gains for YLD and was detrimental to prediction accuracy for TKW (**Supplementary Table 13**).

**Figure 5:**
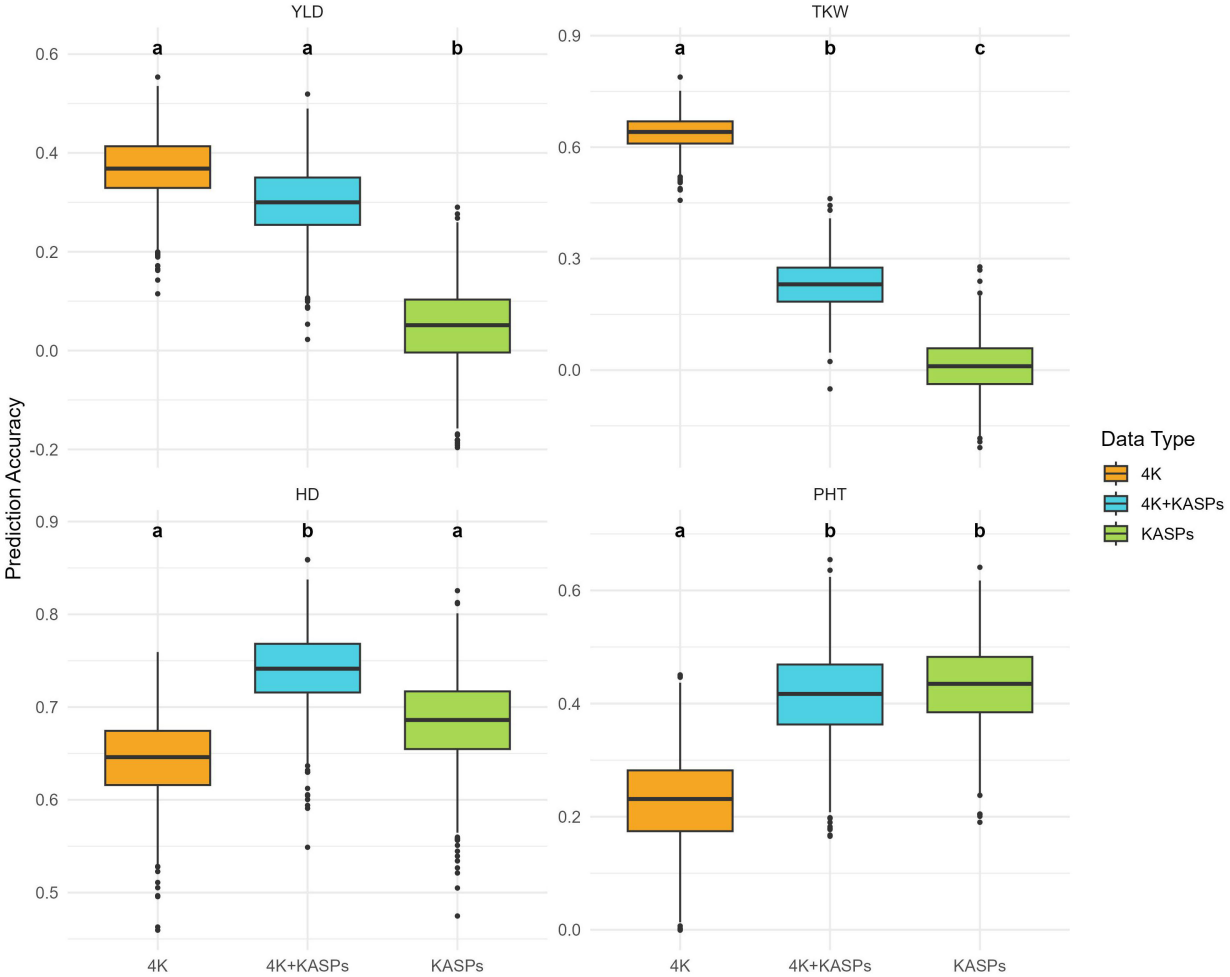
Prediction accuracy distributions for cross-population validation (from GP502 to GP503) using 1,000 bootstrap replicates. The boxplots compare the performance of three marker sets (4K, 4K+KASPs, and KASPs-only) for four traits: Yield (YLD), Thousand-Kernel Weight (TKW), Heading Date (HD), and Plant Height (PHT). Different lowercase letters indicate significant differences (P< 0.05) based on paired bootstrap tests; models sharing a common letter are not significantly different.

Cross-population genomic prediction revealed complex, trait-specific shifts in predictive ability relative to within-population baselines (**Figure 5**, **Supplementary Table 14**). While prediction accuracy for PHT and YLD decreased in the independent validation population, HD and TKW exhibited different trends. The integration of KASP markers as fixed effects provided substantial gains for adaptive traits in this challenging context since the 4K+KASPs model significantly improved prediction accuracy for HD and PHT compared to the SNP-only baseline (**Supplementary Figure 3**). Conversely, adding KASP fixed effects resulted in pronounced reductions in prediction accuracy for YLD and TKW, further highlighting the difficulty of transferring specific marker effects for polygenic traits across diverse genetic backgrounds (**Supplementary Table 15**).

KASP-only models achieved prediction accuracy for HD that matched or exceeded that of high-density SNP-based models (**Figure 4**, **Supplementary Table 12**). For PHT, prediction accuracy obtained using KASP-only models was statistically comparable to that of both low- and high-density SNP panels within the training population (**Figure 4**, **Supplementary Table 13**). This robust performance for adaptive traits was validated in the cross-population context, where KASP-only models maintained their superiority over SNP-only baselines for PHT and remained highly competitive for HD (**Figure 5**, **Supplementary Table 14**). In contrast, KASP-only models produced substantially lower prediction accuracy for YLD (**Figure 5**, **Supplementary Table 15**). These results indicate that KASP-only prediction is effective for traits predominantly controlled by major-effect loci but is inadequate for highly polygenic traits.

Across all analyses, increasing SNP marker density beyond the low-density panel yielded only marginal improvements in prediction accuracy. The largest and most consistent gains were observed when KASP markers were integrated as fixed effects for adaptive traits. Conversely, yield-related traits did not benefit from the fixed-effect strategy, and their predictive performance varied significantly depending on the population context, reflecting their complex genetic architecture. Collectively, these results demonstrate that genomic prediction performance is strongly influenced by interactions among trait architecture, marker strategy, and prediction context.

## Discussion

### 4K versus 90K baseline

A central finding of our within-population analysis is that the 4K panel provided a highly competitive predictive baseline relative to the 90K panel. While trait-specific differences existed, with 90K showing superior accuracy for HD and PHT, and 4K notably outperforming for TKW, the magnitude of these differences was modest (**Supplementary Table 12**). This finding is consistent with the principle of diminishing returns in GS. A systematic evaluation in a large wheat panel by Norman et al. (2018) confirmed that despite finding complex interactions between marker density, population structure, and training set size, the overall response of prediction accuracy to marker density still followed the principle of diminishing returns.

Further insights are provided by the marker subsampling analysis (**Supplementary Figure 4**). These learning curves demonstrate the characteristic pattern of diminishing returns, where predictive ability (*r*) rapidly plateaus at approximately 1,500–2,000 markers (**Supplementary Table 16**). This observation aligns with findings in other small- to medium-sized wheat breeding panels, which also reported that prediction accuracy tends to plateau when marker density reaches approximately 1,000 to 3,000 SNPs (Maulana et al., 2021; Plavšin et al., 2022).

Theoretically, prediction accuracy depends on the linkage disequilibrium (LD) captured between markers and QTLs, as well as the effective number of chromosome segments (*M_e_*) (Meuwissen et al., 2001; Wientjes et al., 2016). When the number of markers (M) substantially exceeds *M_e_*, the independent segments of the genome are already adequately tagged, and continuing to increase marker density yields minimal returns (Wientjes et al., 2016). This principle is strongly supported by our data since the *M_e_* in our population was estimated at around 377 (4K panel) and around 353 (90K panel). The number of markers in our 4K panel (M = 2,600) already far exceeds this estimated *M_e_*. Thus, the full 4K panel performs well because it exceeds the observed saturation point, making the additional markers in the 90K panel (M = 5,004) largely redundant for prediction.

However, these curves also reveal that when subsampled to an equal number of markers (N), the 90K panel consistently showed a slight advantage over 4K for most traits. We attribute this subsampling advantage not to density per se, but to the superior information structure of the 90K marker pool. The 90K array was developed from a deliberately broad discovery space, incorporating diverse accessions from worldwide hexaploid, tetraploid, and wild relative populations to ensure wide applicability and representation (Wang et al., 2014). This difference in origin is critical, as the SNP discovery panel is a primary source of ascertainment bias, which is known to significantly distort population genetic statistics by skewing the MAF spectrum and modifying estimates of linkage disequilibrium (LD) (Albrechtsen et al., 2010; Lachance and Tishkoff, 2013). We hypothesize that the 90K’s broader discovery panel resulted in a more representative MAF spectrum and superior functional coverage. Consequently, a random subset from 90K has a higher probability of capturing informative loci in strong LD with causal variants than a subset from 4K (Geibel et al., 2021).

This baseline comparison also highlighted key trait-specific patterns. For HD and PHT, the 4K panel performed slightly below the 90K panel. This gap is not a fixed limitation and can be effectively bridged by integrating major gene information (KASP). Indeed, both simulation studies and empirical results in wheat demonstrate that incorporating markers linked to known major effect loci as fixed effects into genomic prediction models can significantly increase prediction accuracy (Bernardo, 2014; Rutkoski et al., 2014). This approach is particularly beneficial when the major loci explain more than 10% of the genetic variance and the trait has moderate to high heritability (Bernardo, 2014). This conclusion is consistent with the major roles that known developmental genes, such as those in the *Vrn*, *Ppd*, *FT*, and *Rht* families, play in controlling HD and PHT in wheat (Yan et al., 2004; Beales et al., 2007; Hayat et al., 2019; Brassac et al., 2021; Chen et al., 2022). For YLD, the panels were similar, and its polygenic nature makes it a useful case study for the limits of the KASP strategy (DeWitt et al., 2021; Liu et al., 2022). For TKW, the 4K is better than 90K. We hypothesize that it is driven by sub-genome signal allocation, given that key grain weight genes, such as *TaGW2*, exhibit significant functional differences and interactions among their homoeologs located on the A, B, and D sub-genomes (Simmonds et al., 2016; Zhang et al., 2018).

### Sub-genome signal allocation drives the 4K advantage for TKW

The superiority of the 4K model for predicting TKW may be due to the distinct and complex genetic architecture of this trait. Recent meta-analyses confirm that TKW is influenced by numerous quantitative trait loci (QTL) broadly distributed across the A, B, and D sub-genomes (Tan et al., 2024). Furthermore, its genetic control is known to involve differential contributions from homoeologous gene copies, such as the *TaGW2* family, which show varied effects depending on their specific sub-genome (A, B, or D) location (Simmonds et al., 2016; Zhang et al., 2018; Santantonio et al., 2019). This complex genetic basis provides context for our counter-intuitive observation: while the 90K panel, particularly in equal-N subsampling, demonstrated the benefits of its superior information structure for HD and PHT, the 4K panel consistently outperformed the 90K panel in the within-population baseline prediction for TKW (**Figure 4**; **Supplementary Figure 4**; **Supplementary Tables 12**, **16**).

Our results (see “Effect of Marker Density on Sub-genome Variance Partitioning”) support this hypothesis. The multi-kernel RKHS model used for variance partitioning (**Figure 3**) revealed a significant signal reallocation specifically for TKW. The 4K panel apportioned variance almost equally between the A (0.365) and B (0.364) sub-genomes. In contrast, the 90K panel shifted this weight, reducing the contribution of the A sub-genome (0.296) and increasing the contribution of the B sub-genome (0.428). The direct impact of this reallocation on predictive accuracy is visualized in **Supplementary Figure 5**. In the 4K panel model, the A sub-genome (blue line) serves as the main contributor to overall predictive accuracy. Conversely, in the 90K panel, the predictive signal from the A sub-genome is diminished, resulting in a lower overall accuracy (‘ABD’) compared to the 4K panel. This observation aligns with the principle that differential weighting or contribution of sub-genome additive and interactive effects directly translates into differences in overall predictive performance (Santantonio et al., 2019; Hu et al., 2023). This result highlights that the ‘quality’ of marker information can be as important as the ‘quantity’ of markers.

### Mechanism and density-dependent effects of KASP markers as fixed effects in the training population

Beyond the baseline panel comparison, we evaluated the strategy of incorporating KASP markers as fixed effects (the “+KASP” strategy) within the GP502 training population. This approach yielded highly trait-specific outcomes as it substantially increased prediction accuracy for HD and PHT, offered modest gains for YLD, but was detrimental to TKW (**Figure 4**, **Supplementary Table 12**). This observation aligns with previous studies, which confirm that while incorporating major gene markers as fixed effects can significantly improve prediction accuracy, the efficacy of this approach is highly trait-specific and depends on the functional association between the genes and the trait, as well as the population’s genetic background (Rutkoski et al., 2014; Sarinelli et al., 2019).

The ‘+KASP’ strategy functions by isolating major genetic effects, which may explain why its efficacy is tightly coupled to both the trait’s genetic architecture and the background marker density. The negative impact on TKW, for instance, may be because the selected KASP markers target major phenology and height genes (*Ppd*, *Rht*, *Vrn*, *FT*) which generally have little direct functional association with kernel weight across diverse environments and genetic backgrounds, with reported effects often being indirect or context dependent (Casebow et al., 2016; Velu et al., 2017; Arjona et al., 2018; Liu et al., 2020; Isham et al., 2021; Zhang et al., 2025). Furthermore, kernel weight and size traits are recognized as highly polygenic, controlled by numerous loci with small to moderate effects dispersed throughout the genome, such as *TaGW2* and *TaGS5*, rather than being dominated by the major phenology/height genes targeted by the KASPs (Tillett et al., 2022; Halder et al., 2023). Forcing these indirect major effects into the model likely introduced noise rather than capturing relevant signals for this complex trait.

For traits where KASPs were beneficial (HD and PHT), this success is consistent with their known oligogenic architecture, where a large proportion of the additive genetic variation is often dominated by a few major-effect QTL. Specifically, HD is largely determined by core flowering time genes related to vernalization (*Vrn*), photoperiod (*Ppd*), and flowering integration (*FT/Vrn3*), while PHT is similarly controlled by classic major reduced-height (*Rht*) loci (Zheng et al., 2013; Jobson et al., 2019; Brassac et al., 2021; DeWitt et al., 2021; Isham et al., 2021). Since our KASP markers explicitly target key developmental genes (**Supplementary Table 7**), the approach proved superior for these two traits.

The “+KASP” model effectively deconstructs the phenotypic variance into major, fixed effects captured by KASPs, and the remaining polygenic background captured by the random genetic effect (U) via the RKHS Gaussian kernel. Evidence for this deconstruction is threefold (**Supplementary Figure 6**). First, variance component analysis (**Supplementary Figure 6A**, **Supplementary Table 17**) revealed a significant “shrinkage” in the variance of the random effect (Var(U)) after KASPs were included as fixed effects. This reduction was most pronounced for HD (e.g., -36.6% for 4K) and PHT (e.g., -47.0% for 4K), confirming that the KASPs “absorbed” a large portion of the major genetic variance previously explained by the kernel. This finding is mirrored in other studies. For example, research in maize demonstrated that fitting large-effect SNPs as fixed effects significantly enhanced genomic prediction accuracy, and this improvement was directly related to the corresponding decrease in the estimated genetic variance (Li et al., 2019). This observed “shrinkage” is statistically consistent with the principles of variance partitioning in mixed models. In this framework, fixed effects are designed to explicitly model known, large effects, while the random kernel captures the remaining aggregated polygenic variance (Runcie et al., 2021). By partitioning the major genetic signals to the fixed effects, the variance attributable to the random component (Var(U)) is expected to decrease, as it now only accounts for the residual polygenic background (Yang et al., 2011).

Second, this captured variance was confirmed to be the primary genetic signal, as the explanatory power of PCs on the phenotype dropped precipitously after the KASP effects were removed (e.g., an 88.2% drop for HD-4K; **Supplementary Figure 6B**, **Supplementary Table 18**). This observed drop in PC explanatory power is statistically expected, as the top principal components in genomic data are known to infer and capture the primary axes of genetic variation, which often represents population structure or, in this case, the major loci targeted by our KASPs (Patterson et al., 2006; Price et al., 2006). By fitting these KASP markers as fixed effects, we explicitly modeled and removed this major variance, thus confirming it was the primary signal previously captured by the PCs.

Finally, the ‘incremental’ nature of this strategy is demonstrated by the varying predictability of the phenotype residuals (**Supplementary Figure 6C**, **Supplementary Table 19**). For HD, the residual genomic selection accuracy dropped to a low level (*r* = 0.188 for 4K, and 0.147 for 90K), indicating that the KASP markers had already exhausted most of the predictive genetic signal, leaving little for the background kernel to capture. In contrast, for TKW, the residual prediction accuracy remained high (*r* = 0.430 for 4K, and 0.401 for 90K), confirming that the primary genetic drivers for kernel weight reside in the widespread polygenic background rather than the targeted major loci.

This mechanism’s effectiveness is also highly dependent on the background marker density (**Supplementary Figure 7**). The prediction gain (Δr) from the +KASP strategy exhibited a clear pattern of diminishing returns as the number of background markers (N) increased. This complements our earlier finding that baseline GS accuracy plateaus around 1,500–2,000 markers. As the background panel becomes dense enough to capture the LD of major QTLs, the marginal utility of adding those QTLs explicitly as KASPs decreases. This density-dependence directly explains our baseline observations. For HD, the 90K panel was initially superior to the 4K (Δ=+0.069). However, the +KASP strategy provided a far greater boost to the 4K panel (Δr=+0.179) than to the 90K (Δr=+0.099) (**Supplementary Table 12**). This asymmetrical gain not only bridged the performance gap but even resulted in the 4K+KASP model (r= 0.592) slightly outperforming the 90K+KASP model (r= 0.581). This boosting effect was further validated in the cross-population context. For PHT, where the baseline 4K panel’s cross-population accuracy was particularly low (r = 0.227), incorporating KASPs as fixed effects raised the prediction level to 0.414 (**Supplementary Table 14**). Therefore, integrating major gene information via KASPs enhances the predictive power of low-density panels, precisely for traits where those panels fail to fully capture major, known genetic effects.

### Predictive utility of KASP markers and the transferability of their effects

The KASP marker set, comprising a small number of diagnostic loci, demonstrated differences in its predictive utility depending on population context. In the within-population context (GP502), the performance of the KASP-only model showed divergence across traits (**Supplementary Table 12**). It demonstrated strong predictive ability for HD (r=0.564) and PHT (r=0.553) but was substantially less effective for TKW (r=0.133). Unexpectedly, it also achieved a moderate level of accuracy for YLD (r=0.418). This divergence in predictive ability was mirrored by the within-population stability of the KASP marker effects, since these effects were estimated to be highly robust for HD (cosine=0.946) and PHT (cosine=0.955), moderately robust for YLD (cosine=0.888), but significantly less stable for TKW (cosine=0.553) (**Supplementary Figure 8A**).

The strong performance for HD and PHT aligns with findings in biparental populations where the oligogenic architecture of such traits means that simple QTL-based models can be sufficient for accurate prediction and may even outperform standard polygenic genomic selection models (DeWitt et al., 2021). Our results confirm this principle holds true for a diversity panel. For HD, the KASP-only model (r=0.564) significantly outperformed both the 4K (r=0.413) and the 90K (r=0.481) baseline panels. Similarly, for PHT, its ability (r=0.553) was superior to the 4K baseline (r=0.525) and statistically comparable to the 90K baseline (r=0.561) (**12**). In contrast, the model’s poor performance for TKW is likely attributable to the fact that the selected KASP markers (targeting *VRN*/*PPD*/*FT*/*Rht* loci) are not directly associated with this trait (Casebow et al., 2016; Velu et al., 2017; Arjona et al., 2018; Liu et al., 2020; Isham et al., 2021; Zhang et al., 2025), and that kernel weight and size are recognized as being highly polygenic (Tillett et al., 2022; Halder et al., 2023). While the KASP-only model showed moderate accuracy for YLD within the training population, this performance was not robust, collapsing in the cross-population tests.

When applied to the more challenging cross-population scenario, the model’s utility remained highly trait-dependent, largely extending the trend observed within the GP502 population. Predictive ability stayed strong for HD (r=0.683) and PHT (r=0.431) and was negligible for TKW (r=0.01). However, a significant divergence occurred for YLD. Its accuracy plummeted from r=0.418 in the within-population context to just r=0.051 for cross-population. An analysis of marker effect stability and transferability directly supports this divergence (**Supplementary Figure 8**). For PHT and HD, which remained predictive, marker effects showed high cross-population cosine consistency (0.891 and 0.826, respectively; **Supplementary Figure 8B**) and strong to moderate rank correlation between populations (ρ=0.74 and ρ=0.50; **Supplementary Figure 8C**). This combination of consistency and correlation confirms their effects are robust and transferable. Conversely, TKW, which failed in both contexts, showed extremely low stability (cosine=0.303) and transferability (ρ=0.11). YLD presents a more complex case. While its marker effects appeared relatively stable (cosine=0.757) and moderately correlated (ρ=0.54), its predictive accuracy was negligible (r=0.051). This critical finding suggests that while the model’s parameter estimates are statistically robust, the loci themselves (*VRN*/*PPD*/*FT*/*Rht*) lack predictive power for YLD in the elite GP503 population.

Beyond Pearson’s r, prediction-error and calibration metrics further characterized cross-population performance (**Supplementary Table 21**). For YLD, all models showed pronounced mean under-prediction, consistent with the yield difference between the diverse training set and the elite validation population. After removal of this mean offset, centered RMSE was lower for 4K and 4K+KASPs than for KASP-only. The 4K calibration slope for YLD was close to 1 (1.10), indicating well-calibrated predictions, whereas the KASP-only slope was 0.18. For TKW, 4K had the lowest centered RMSE, although its slope was 1.97. The KASP-only model had essentially no predictive signal for YLD or TKW. For HD, 4K+KASPs had the lowest centered RMSE and highest r (**Supplementary Tables 14**, **21**). For PHT, the centered RMSE of KASP-only was close to 4K. Together, these metrics reinforced the trait-dependent utility of KASP markers: KASP-only models retained useful predictive signal for adaptive traits governed by major-effect loci but showed limited calibration and were unreliable for polygenic yield and grain-weight traits.

This failure is likely driven by a germplasm-class mismatch between the training and validation sets. Our KASP set targeted key adaptation loci (*VRN*/*PPD*/*FT*/*Rht*). The training set (GP502) is an intentionally diverse panel, whereas the validation set (GP503) consists of elite lines pre-selected within a single breeding program. This contrast in diversity and relatedness can alter allele frequencies at these loci and, consequently, the portability of their effects. In an elite validation set like GP503, these adaptive alleles are often constrained by prior regional selection, as breeding programs historically favor specific alleles, such as the photoperiod-insensitive *Ppd-D1a* (Andeden et al., 2011; Kiss et al., 2014; Lozada et al., 2021). This selection can lead to limited segregating variation or near-fixation (Kiss et al., 2014), a trend observed in our data as markers for *Vrn-B1* and *Ppd-D1* showed reduced diversity in GP503 compared to GP502 (**Supplementary Table 20**). Under such allele-frequency shifts, the fixed-effect KASP terms carry little predictive variance in the target set, even if their estimated effects are statistically stable (as seen for YLD). Cross-population accuracy is known to erode when QTL MAFs, LD patterns, or segregation status differ between training and target populations (Wientjes et al., 2015; Schopp et al., 2017).

## Conclusion

This study shows that increasing SNP density beyond the 4K panel provided limited overall gains in genomic prediction, although the relative performance of the 4K and 90K panels varied among traits. The integration of KASP markers as fixed effects significantly enhances prediction accuracy for adaptive traits such as HD and PHT, demonstrating the value of explicitly modeling major-effect loci within genomic selection frameworks. These results provide a practical basis for reducing genotyping costs in breeding programs without sacrificing selection accuracy for traits with well-characterized genetic architecture. The strong performance of KASP-only models for these traits further indicates that ultra-low-cost, targeted genotyping can support early-generation selection when population sizes are large. In contrast, the limited predictive ability observed for yield-related traits confirms that improvement of highly polygenic traits remains dependent on genome-wide marker information. Together, these findings establish a trait-informed genotyping strategy that enables breeding programs to balance cost and predictive performance, thereby enhancing the efficiency and scalability of genomic selection under operational breeding conditions.

## Data Availability

The data presented in the study are deposited in the Zenodo repository under record 20401810, DOI: 10.5281/zenodo.20401810 (Gao et al., 2026).

## Supplementary material

Supplementary Figures 1–8 Additional figures showing population structure, prediction accuracy distributions, etc. (Supplementary_Figures.docx).

Supplementary Tables 1–21 Detailed supporting data including population information, marker statistics, and full prediction accuracy results. (Supplementary_Tables.xlsx).
