# Supplementary material for: Genomic prediction using a cost-effective mid-density SNP platform in spring wheat
Source: Front Plant Sci. 2026 Jul 20;17:1892246. doi: 10.3389/fpls.2026.1892246 (PMC13429604; doi:10.3389/fpls.2026.1892246)
Supplement: Supplementary Figures 1–8 — Additional figures showing population structure, prediction accuracy distributions, etc. (Supplementary_Figures.docx). [file DataSheet1.docx]

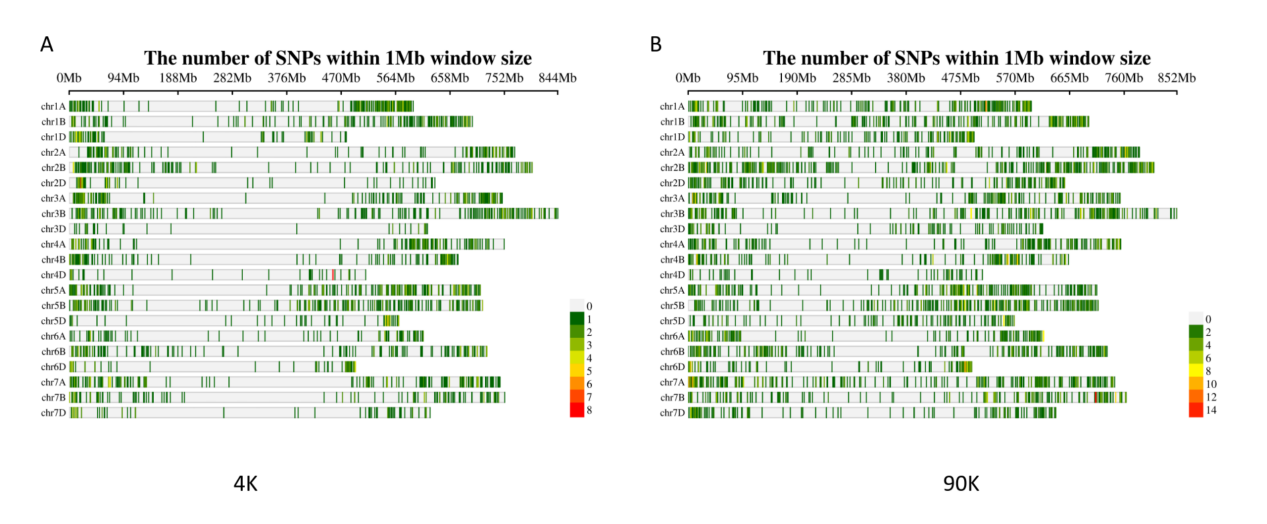


**Supplementary Figure 1** Comparison of SNP marker distribution across the 21 wheat chromosomes for the 4K and 90K panels. The number of SNPs is plotted for each chromosome, which are ordered and grouped by sub-genome (A, B, and D), as indicated by the color-coding. The figure illustrates the genome-wide coverage and highlights the broadly concordant distribution patterns between the two arrays, such as the higher marker density on the B genome and lower density on the D genome.


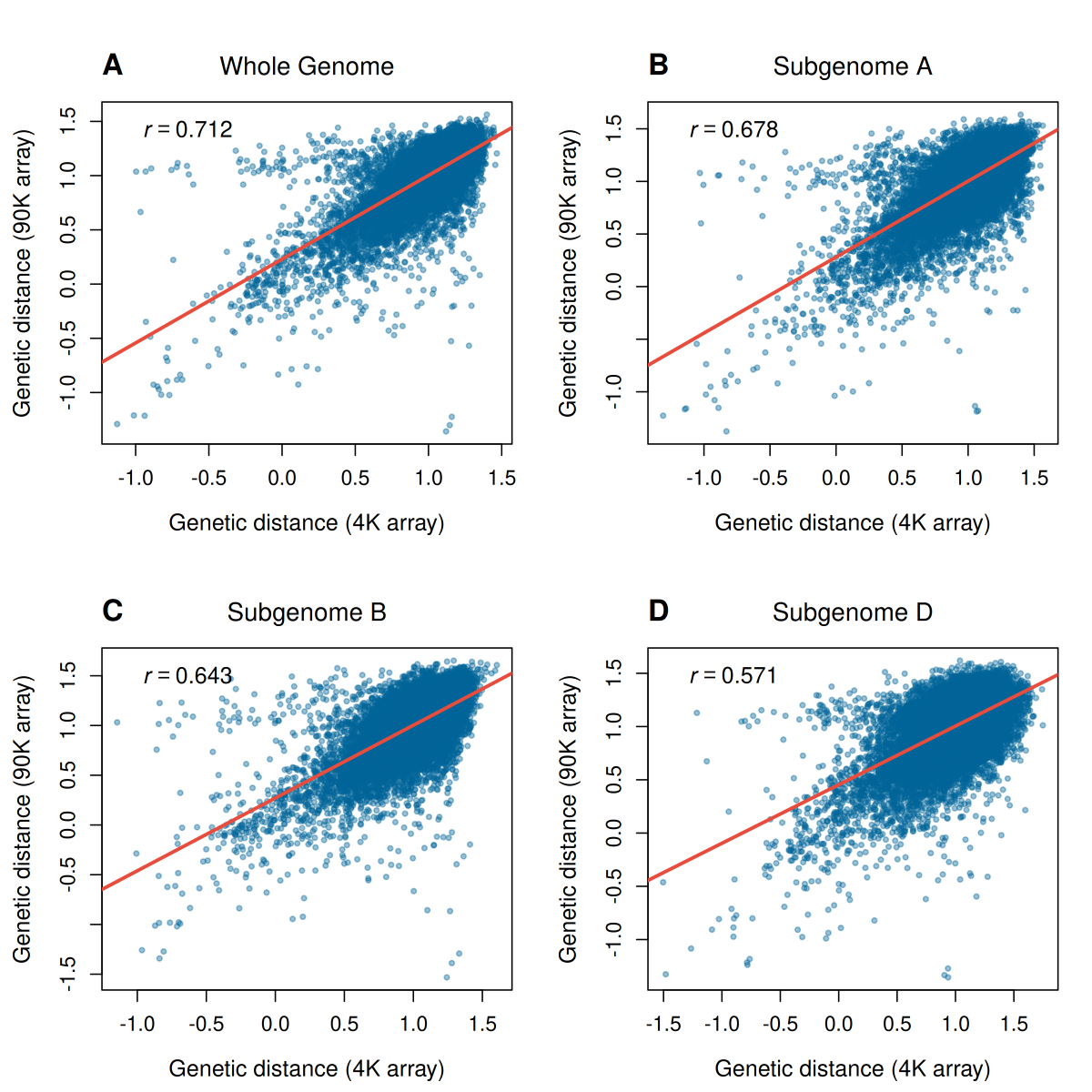


**Supplementary Figure 2 Correlation of genetic distances between 4K and 90K marker panels.** Scatter plots showing the correlation of genetic distances (kinship values) derived from the 4K array versus the 90K array for (A) the whole genome, (B) Subgenome A, (C) Subgenome B, and (D) Subgenome D. The red line represents the linear regression, and 'r' denotes the Pearson correlation coefficient (all P < 0.001).


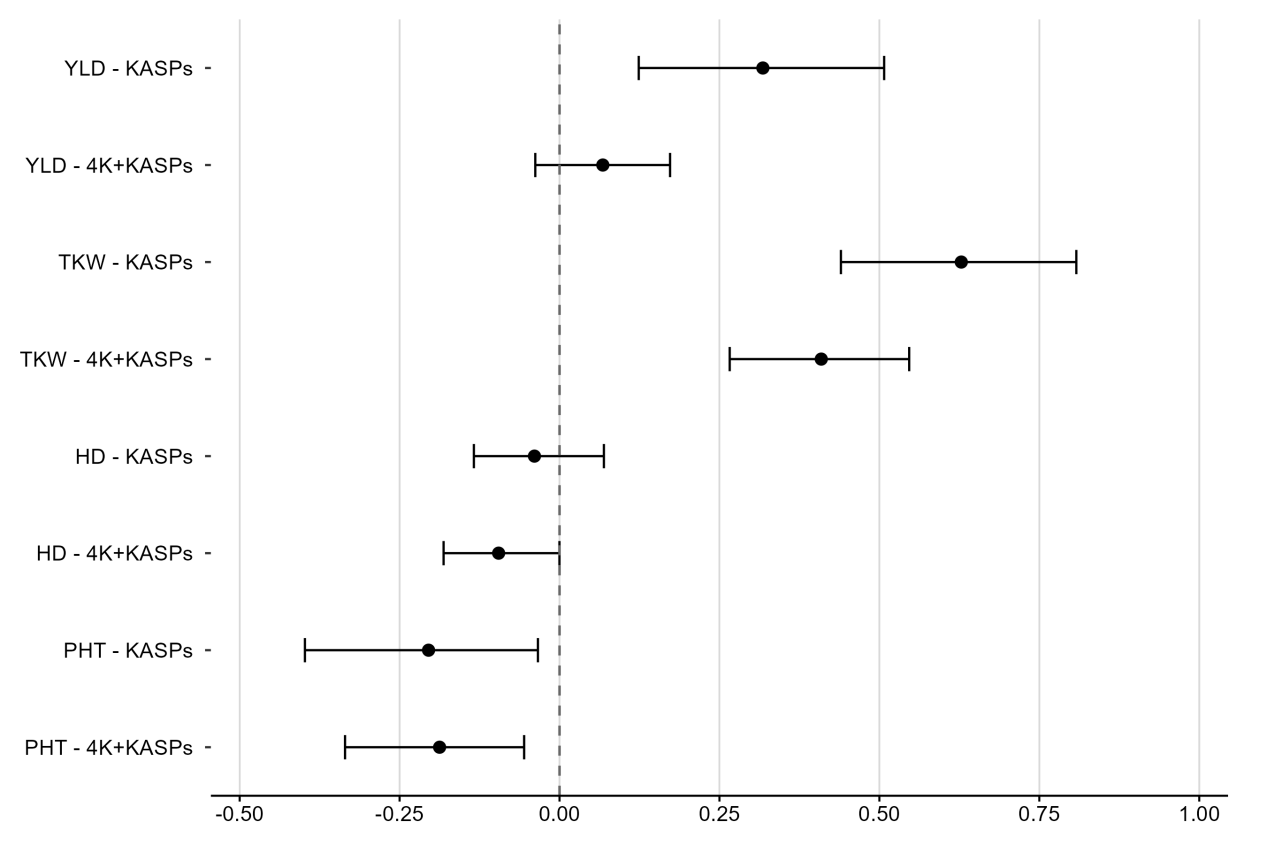


**Supplementary Figure** **3** Forest plot of paired accuracy comparisons for the cross-population validation from GP502 to GP503, based on 1,000 bootstrap replicates. The plot visualizes the mean difference (Δ, points) and 95% confidence intervals (bars) for two comparisons against the 4K baseline: 4K+KASPs vs. 4K and KASPs-only vs. 4K. The vertical dashed line at Δ=0 represents no change in accuracy. Points to the right indicate an improvement over the 4K baseline, while points to the left indicate a decrease.


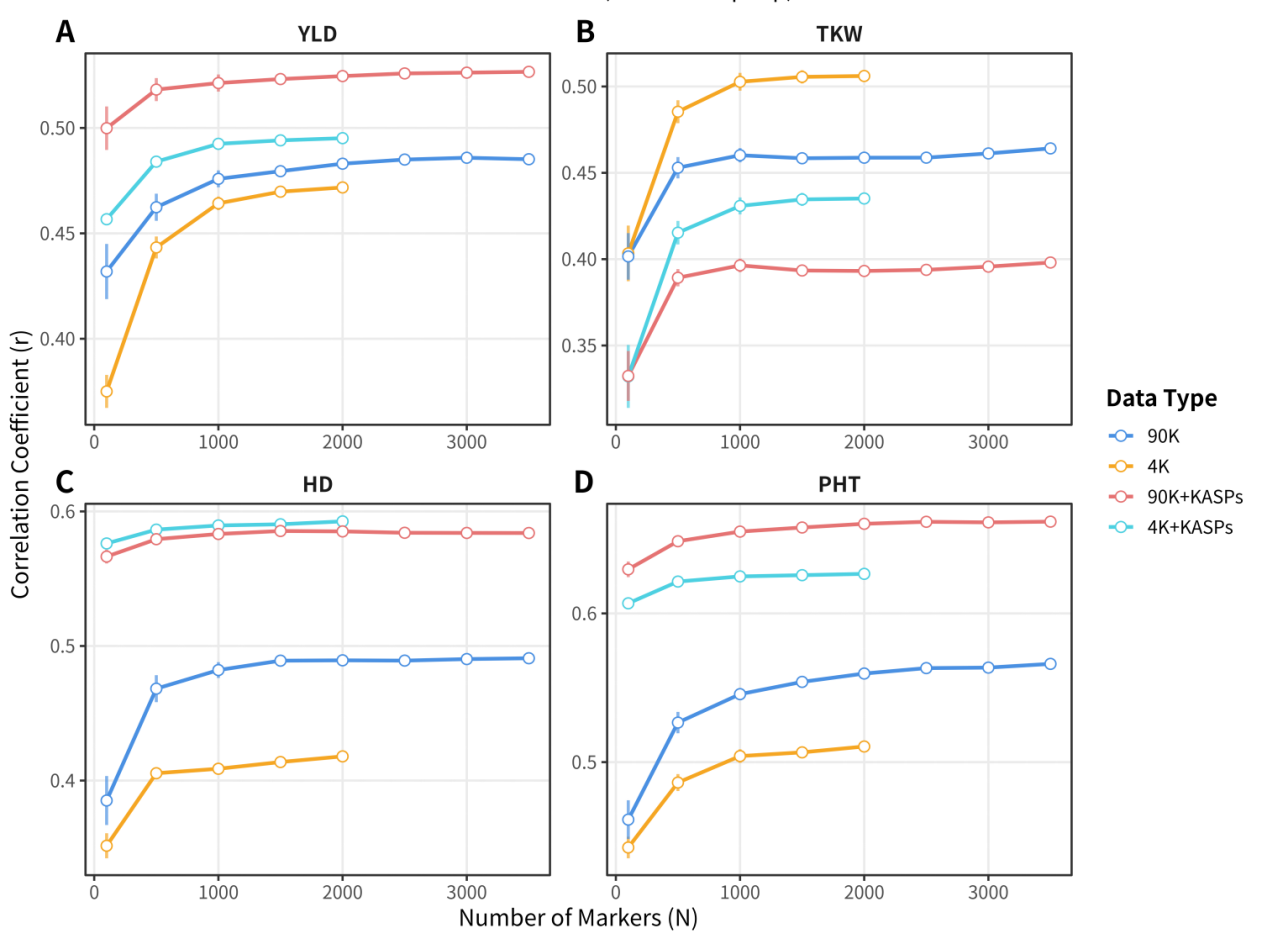


**Supplementary Figure 4** Learning curves for genomic prediction accuracy based on marker subsampling. Mean genomic prediction accuracy (y-axis) plotted against the number of markers (N) subsampled (x-axis) for the 4K and 90K panels in the within-population analysis. Curves are shown for four traits: heading date (HD), plant height (PHT), yield (YLD), and thousand-kernel weight (TKW). Data points represent the mean accuracy derived from 10 replications of random marker subsampling. Error bars indicate the standard error of the mean (SE). The curves illustrate a pattern of diminishing returns, with prediction accuracy typically reaching a plateau after 1,500–2,000 markers


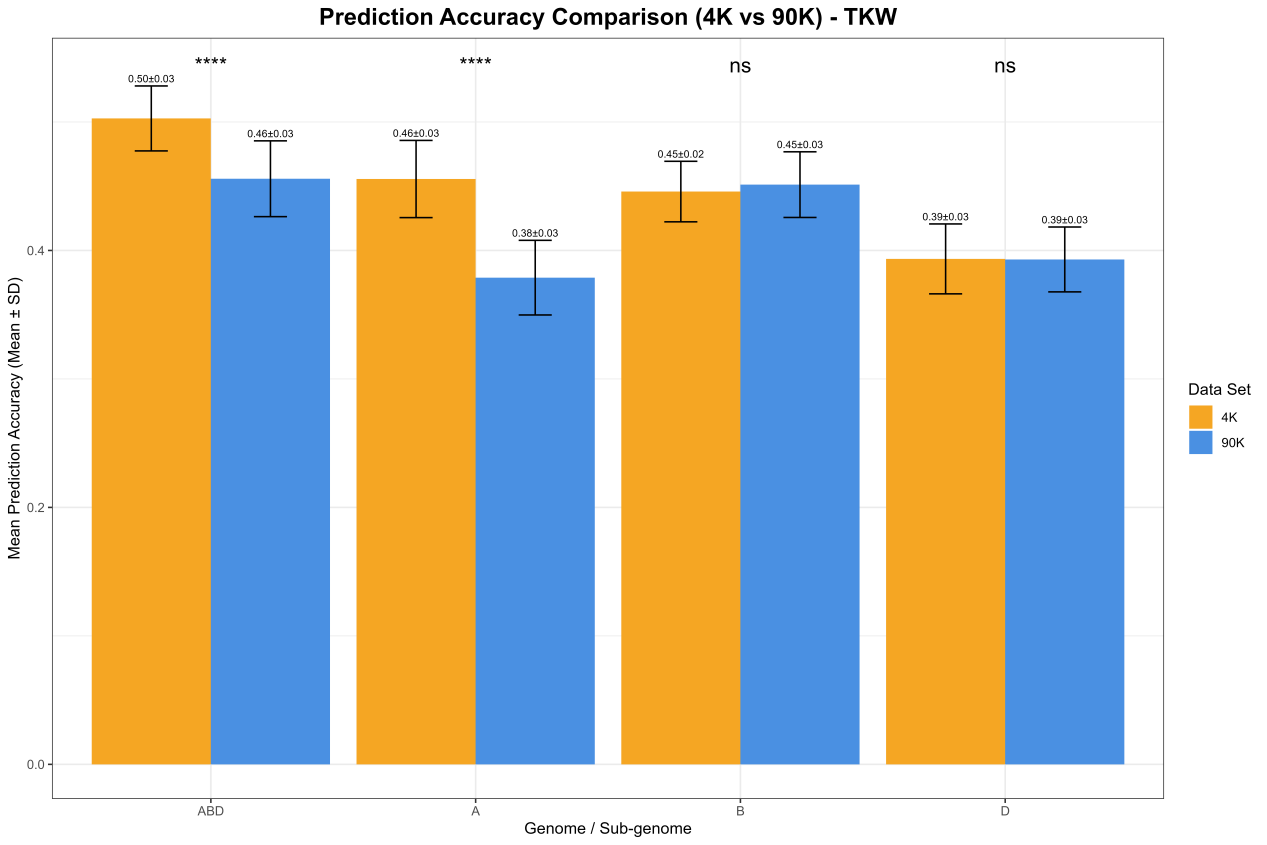


**Supplementary Figure 5** Subgenome contributions to TKW genomic prediction accuracy for the 4K and 90K panels. “Genomic prediction accuracy (y-axis) for thousand-kernel weight (TKW) using a multi-kernel RKHS approach, plotted against the subsampling proportion of the training population (x-axis). The analysis compares the 4K panel (left) and the 90K panel (right). Lines represent the predictive abilities of models built using: A-subgenome markers only ('A'), B-subgenome markers only ('B'), D-subgenome markers only ('D'), and the full SNP model integrating all three subgenomes ('ABD'). This visualization highlights the shift in signal contribution: the A subgenome ('A') is the dominant predictor in the 4K panel, whereas its contribution is diminished in the 90K panel and replaced by the B subgenome ('B'). Notably, the overall accuracy of the full model ('ABD') is higher for the 4K panel.


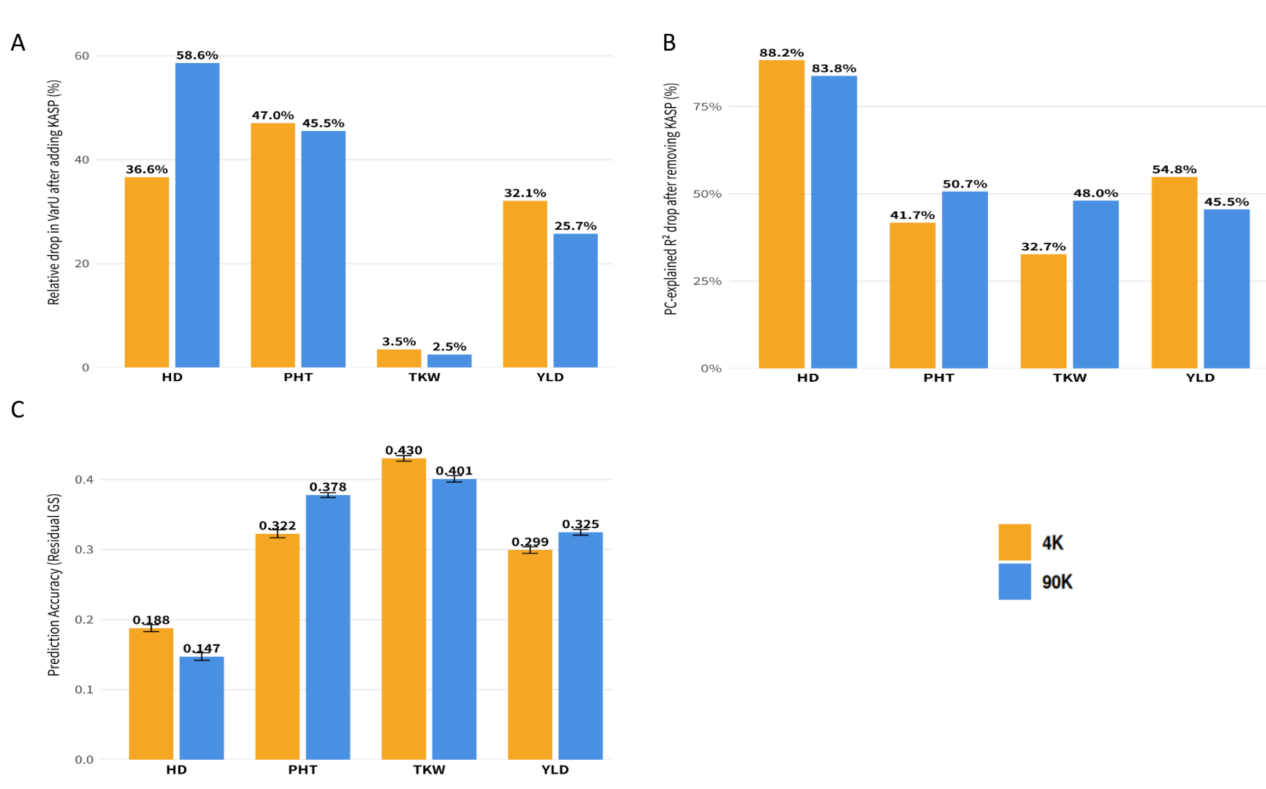


**Supplementary Figure 6** Mechanism of the KASP markers as fixed effects strategy in the GP502 training population (A) Comparison of the random genetic effect variance (Var(U)) between the baseline model (without KASP) and the KASP markers as fixed effects strategy (+KASP model). The inclusion of KASPs as fixed effects leads to a significant "shrinkage" of the random variance component (Var(U)), particularly for HD and PHT, indicating the KASPs successfully absorbed major genetic variance previously captured by the kernel. (B) Genomic prediction accuracy of the "Residual-GS" model, where the effects explained by the 13 KASPs were first removed from the phenotype. The remaining, trait-dependent predictability (e.g., low for HD, higher for PHT and TKW) demonstrates the presence of a polygenic background independent of the major KASP-QTLs. (C) Reduction in the coefficient of determination ($\text{R}^{\text{2}}$) from a model regressing the phenotype onto the top 10 genomic Principal Components (PCs) (derived from either the 4K or 90K panel). The baseline $\text{R}^{\text{2}}$($\text{R}_{\text{y}\text{\_}\text{on}\text{\_}\text{PCs}}^{\text{2}}$) is calculated using the original phenotype. After removing the fixed effects explained by KASPs, the $\text{R}^{\text{2}}$ is recalculated by regressing the remaining phenotypic residuals onto the same 10 PCs ($\text{R}_{\text{resid}\text{\_}\text{on}\text{\_}\text{PCs}}^{\text{2}}$). The substantial drop confirms that the KASP markers captured the primary genetic signals associated with major population structure, which were initially explained by the top 10 PCs.


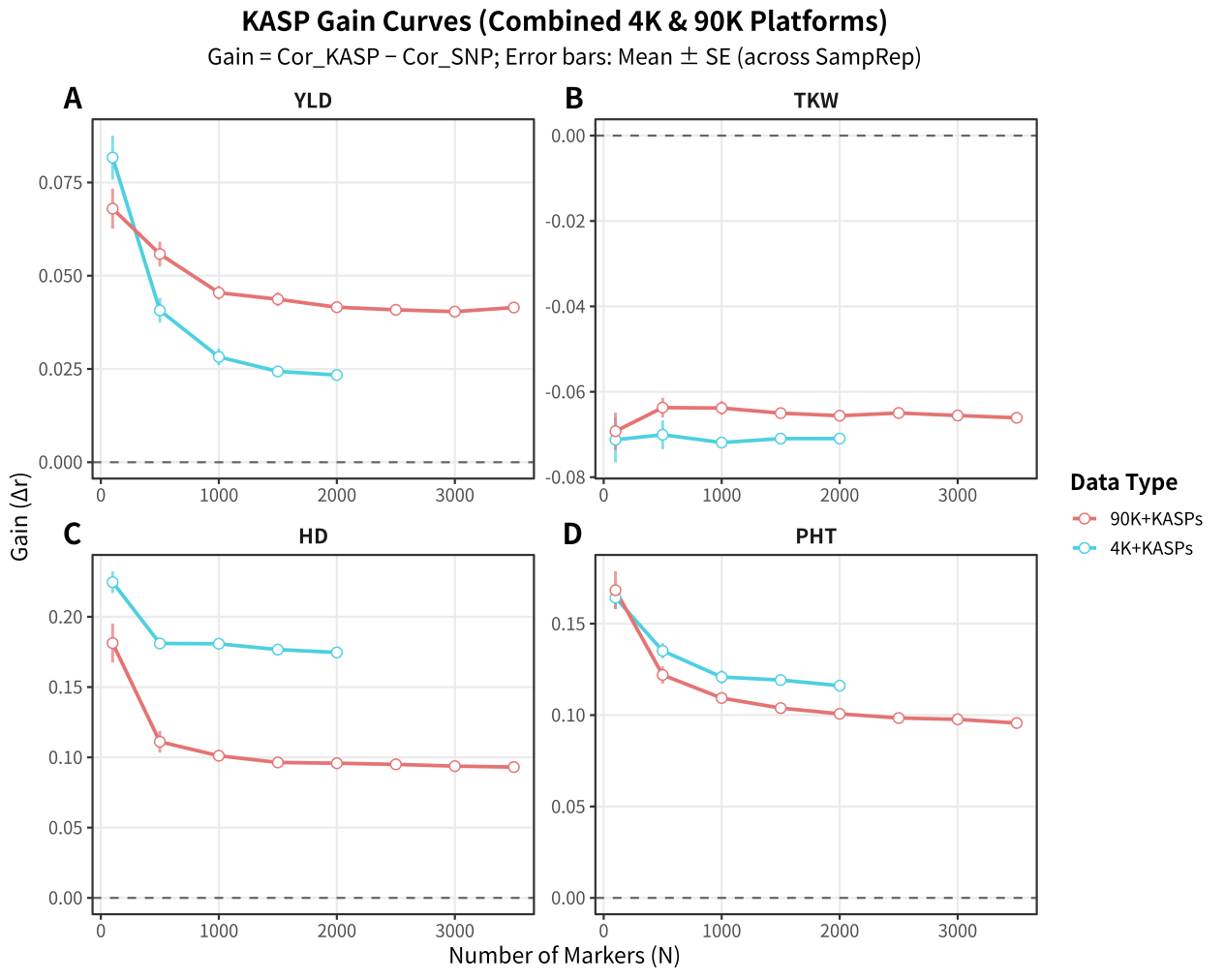


**Supplementary Figure 7** Gain in prediction accuracy (Δr) from the +KASP strategy as a function of background marker density. The plot shows the mean gain in prediction accuracy (Δr) achieved by adding 13 KASP markers as fixed effects (+KASP) to the baseline RKHS model within the GP502 population. The x-axis represents the number of background markers (N) sub-sampled from either the 4K (e.g., blue lines) or 90K (e.g., red lines) panels. The gain on the y-axis (Δr) is calculated as the difference in accuracy between the +KASP model and the baseline model at each corresponding marker density N (r_+KASP_ − r_Baseline_). Curves are shown for the four traits: Heading Date (HD), Plant Height (PHT), Yield (YLD), and Thousand-Kernel Weight (TKW). Shaded areas represent the standard error of the mean across 10 sub-sampling replications. The graph illustrates that the marginal benefit of the +KASP strategy is highest at low marker densities and exhibits diminishing returns as the background panel becomes denser


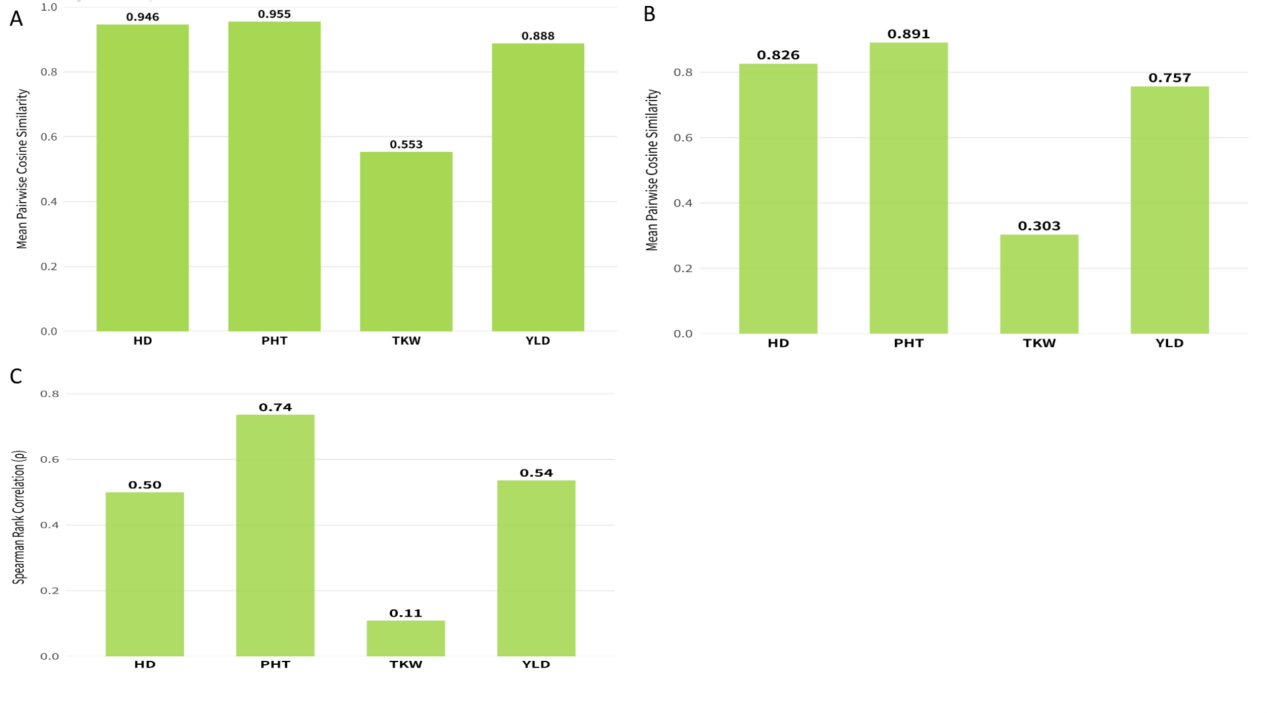


**Supplementary Figure 8** Stability and cross-population transferability of KASP marker effect estimates. (A) Within-population cosine consistency of KASP marker effects, estimated from 1000 bootstrap replicates within the GP502 training population. (B) Cross-population cosine consistency, assessing the stability of effect estimates derived from 1000 bootstrap replicates of the GP502 training set when applied to the GP503 validation set. High cosine consistency values (near 1.0) in both (A) and (B) indicate that the model's parameter estimates are stable and robust. (C) Spearman rank correlation (ρ) comparing the final KASP marker effect estimates derived independently from the full GP502 dataset versus the full GP503 dataset. This metric quantifies the transferability (i.e., conservation of effect rank and direction) of marker effects between the two populations. The high correlation for PHT (ρ=0.74) and moderate correlation for HD (ρ=0.50) suggest conserved effects, while the low correlation for TKW (ρ=0.11) indicates that its KASP marker effects are unstable and not transferable.
